# Physical Activity and Quality of Life in Adult Men and Women with Distorted Perception of Weight Status: Nationwide Surveys (KNHANES 2016–2018)

**DOI:** 10.3390/ijerph191610417

**Published:** 2022-08-21

**Authors:** Su-Jin Lee, Min-Gyu Lim, Jung hee Kim, Chulyong Park, YoungJi Ko, Myung-Gwan Kim, Chul-Hyun Kim, Aeryoung Kim, Jong-Moon Hwang

**Affiliations:** 1 Graduate School of Public Health, Kyungpook National University, Daegu 41944, Korea; 2 Department of Rehabilitation Medicine, Kyungpook National University Hospital, Daegu 41944, Korea; 3 College of Pharmacy, Industrial Pharmacy, Chungbuk National University, Cheongju-si 28644, Korea; 4 Department of Occupational and Environmental Medicine, Yeungnam University Hospital, Daegu 42415, Korea; 5 Department of Preventive Medicine and Public Health, College of Medicine, Yeungnam University, Daegu 42415, Korea; 6 Department of Nursing, Daegu Hanny University, 1 Hanuidae-ro, Gyeongsan-si 38610, Korea; 7 Department of Biomedical Informatics, CHA University School of Medicine, Seongnam 13415, Korea; 8 Institute for Biomedical Informatics, CHA University School of Medicine, Seongnam 13415, Korea; 9 Department of Rehabilitation Medicine, School of Medicine, Kyungpook National University, Daegu 41566, Korea

**Keywords:** body image perception, subjective body recognition (SBR), physical activity, quality of life, distorted perception of weight

## Abstract

This study analyzed the distorted perception of weight in adults aged 20 years or older and investigated the difference in the amount of physical activity and health-related quality of life (HRQOL). This study examined 21,326 adults regarding their body mass index (BMI), subjective body recognition (SBR), physical activity (according to the Global Physical Activity Questionnaire; GPAQ), and HRQOL (EuroQol-5 Dimension; EQ-5D) from the 7th Korea National Health and Nutrition Examination Survey (2016–2018). Independent *t*-test, analysis of variance (ANOVA), chi-square test, and multiple regression analysis were conducted. The group with the same BMI and SBR significantly showed a long time of activity vigorous-intensity (F = 21.25, *p* = 0.003) and moderate-intensity time (F = 17.24, *p* < 0.001). In the ‘BMI = SBR’ group, the sub-group with normal BMI and normal SBR showed the highest vigorous-intensity (mean ± SD = 7.20 ± 26.05, F = 37.86, *p* < 0.001) and moderate-intensity (mean ± SD = 13.89 ± 30.18, F = 43.27, *p* < 0.001) activity times. The sub-group with normal BMI and normal SBR had the highest percentage of responding as normal in the five EQ-5D sub-items. For the score of the EQ-5D Index, the highest score was shown in the group that felt subjectively more obese than the actual BMI (F = 56.83, *p* < 0.001). In the ‘BMI = SBR’ group, these factors related to physical activity (vigorous-intensity, moderated-intensity, and walking) are factors influencing health-related quality of life in this regression model (F = 396.57, *p* < 0.001, R^2^ = 0.165). Various health promotion programs and policy recommendations to reduce the distorted perception of weight are required.

## 1. Introduction

Obesity, previously considered a problematic phenomenon only in developed western countries, is identified by the World Health Organization as a global epidemic with considerable public health issues [1]. It may persist into greater health issues due to increased risk of chronic diseases, such as heart disease, diabetes, hypertension, cerebral stroke, and various types of cancers [2]. Therefore, the understanding of obesity is pertinent and bears immense public health implications [3].

Although its prevalence has been increasing, societal ideals of cosmetic beauty have continued to emphasize thinness and leanness [4]. Such cultural stress has recently resulted in an eagerness to try various weight loss strategies and immense body preoccupation [5]. Body weight is determined by individual genetics and environmental and cultural factors [6]. Furthermore, among these identified predisposing contributors, social elements, such as diet and physical activity, are controllable risk factors [7]. In an attempt to reduce the rise of the prevalence of obesity, many public health movements have strived to promote healthier lifestyles [8]. Diverse campaigns have aimed to increase physical activities and reduce calorie intake [9,10].

The perception of body image plays an important role in body weight management [11]. “Body image” is defined as one’s subjective internal representation of their outer appearance [12]. Its construct is multidimensional and the product of individual perceptions, feelings, prior and present experiences, values, beliefs, attitudes, and societal expectations [12]. Body image also varies according to age and gender, reflecting the aging process and differences in social gender roles [13]. Thus, an investigation on body image in diverse subpopulations is necessary. Negative body image perception is common and has deleterious physical effects and emotional distress on all ages and genders [14,15].

With society and media making slim figures the standard of health and beauty, a discrepancy between one’s actual body size and subjective body perception is also rising, causing damaging outcomes [16]. Specifically, body image disturbance is known to lead to lower self-esteem, depression, eating disorders, social anxiety, and dramatic measures to alter one’s external appearance [16,17,18].

Sober assessment of body image in relation to actual body size is essential to better understand its relationship with physical activity and quality of life [19,20]. Since there are damaging consequences associated with body image discrepancy and its malleable and subjective nature, the general population would benefit by better understanding the relationship between body image, actual body size, and any subsequent behaviors this discrepancy may cause [20].

In healthy people who do not have psychological problems, distorted body images may indicate low quality of life [21]. Lee et al. [21] investigated weight control practices, self-image perception, and quality of life of adults with distorted body images. In this study, among the study subjects corresponding to the low quartile of quality of life, those who recognized that excessive weight control was necessary due to distorted body perception showed lower quality of life in mental health, daily life, sexual relations, and work–process balance. This is consistent with several studies [22,23] stating that distorted body perception reflects negative psychological states such as unhealthy behavior and diet, low self-esteem, and suicidal thoughts, and was confirmed as an influencing factor for low quality of life.

Regarding previous studies, most research subjects were adolescent females or university students. Furthermore, research on body image perception and actual health outcomes according to appearance usually included female patients who underwent a mastectomy for breast cancer. Thus, comparative studies targeting entire general populations are insufficient. Hence, the results cannot be generalized due to the limited number of subjects. In addition, the report reporting the effect of distorted perception of weight on quality of life was conducted on patients who underwent surgery, but studies on the general adult population were insufficient [24,25,26].

This study aimed to analyze the discrepancy between the actual level of obesity according to body mass index (BMI) and subjective body recognition, by comparing a large Korean general population, from the Korean National Health Survey and analyzing the difference in physical activity performance time according to the distorted perception of weight. Further, this study also investigated the difference in health-related quality of life according to the distorted perception of weight. The results could be used as basic data for effective and practical weight control methods provided by healthcare professionals.

An analysis of the factors affecting quality of life can serve in various ways as the general population engages in daily life. Suggestions can be made to improve general health outcomes by identifying the social and cultural factors that contribute to negative health outcomes in the general population. This may prevent excessive economic health spending.

## 2. Methods

### 2.1. Study Population and Data Collection

This study is based on the 7th Korea National Health and Nutrition Examination Survey (KNHANES) 2016–2018 [27]. The description of the study was prepared according to the STROBE reporting guidelines (https://www.strobe-statement.org/, accessed on 8 August 2021). The KNHANES is a nationwide, population-based cross-sectional health survey performed by the Korea Disease Control and Prevention Agency. The KNHANES calculates nationally representative and reliable statistics on the health level, health behavior, and food and nutritional intake of the Korean people [27]. The National Health and Nutrition Examination Survey uses weighted data analysis to increase the representativeness and accuracy of estimates related to people’s health behavior, chronic disease prevalence, and nutritional intake. The basic weights of the 7th period data were composed of health questionnaire, examination weight, and nutrition survey weight [27].

Adults aged 20 years or older, including men and women, were extracted from the subjects of the National Health and Nutrition Examination Survey conducted in 2016–2018 by the stratified sampling method for all Koreans. The final analysis subjects of this study were 21,326.

The general characteristics included sex (male or female), age (20–39 year, 40–64 year, ≥65 year), annual household income (high: ≥75,000 dollar (converting Korean currency to USD), middle-high: USD ≥ 45,000, middle-low: USD ≥ 20,000, or low: USD < 20,000, education level (≥college, high school, middle school, or ≤elementary school).

### 2.2. Body Mass Index (BMI) × Subjective Body Recognition (SBR)

Body mass index (BMI) and subjective body recognition (SBR) were classified according to the National Health and Nutrition Examination Survey Data guidelines (BMI: normal-obesity-underweight and SBR: normal-obesity-underweight). In the National Health and Nutrition Survey, body weight and height were collected through physical measurement, and BMI was analyzed through the collected measurement data. In the collected BMI index, those with a BMI of less than 18.5 kg/m^2^ are classified as ‘underweight’, those with a BMI index of 18.5 kg/m^2^ or more and less than 25 kg/m^2^ are classified as ‘normal’, and those with a BMI of 25 kg/m^2^ or more are classified as ‘obesity’.

SBR was collected through a health survey. The responses to SBR were ‘very underweight’, ‘underweight’, ‘normal’, ‘slightly obese’, and ‘very obese’. In our study, among the response to SBR, ‘very underweight’ and ‘underweight’ were classified as ‘underweight’, ‘normal’ was classified as ‘normal’, and ‘slightly obese’ and ‘very obese’ were classified as ‘obesity’.

In this study, the status showing the difference between body image recognition and actual weight is expressed as the distorted perception of weight, and the sub-groups are as follows:

Category 1. BMI = SBR: BMI (normal) × SBR (normal), BMI (obesity) × SBR (obesity), BMI (underweight) × SBR (underweight).

Category 2. BMI < SBR: BMI (normal) × SBR (obesity), BMI (underweight) × SBR (obesity), BMI (underweight) × SBR (normal).

Category 3. BMI > SBR: BMI (normal) × SBR (underweight), BMI (obesity) × SBR (normal), BMI (obesity) × SBR (underweight).

### 2.3. Physical Activity: Global Physical Activity Questionnaire (GPAQ)

In the Korean National Health and Nutrition Examination Survey before 2014, the International Physical Activity Questionnaire (IPAQ) was used as the physical activity recall questionnaire to measure the amount of physical activity [28]. Since 2014, the Korea National Health and Nutrition Examination Survey has investigated the physical activity of the population by using the GPAQ in order to accurately measure and interpret the amount of physical activity by area (activity at work, travel to and from places, and recreational activities) as well as considering the intensity of physical activity [27]. GPAQ [29] was translated into Korean by the Korea Disease Control and Prevention Agency in 2013 and its reliability and validity were verified [30]. In this study, time was used to answer questions for each physical activity intensity (vigorous-intensity activities, moderate-intensity activities). For each of the three domains (activity at work, travel to and from places, and recreational activities), time was calculated according to vigorous-intensity and moderate-intensity activities (the numbers of days of activity per week × number of times (min) of activity per day).

The definition of ‘Vigorous-intensity activities’ in GPAQ used in the 7th Korea National Health and Nutrition Examination Survey is ‘activities that require hard physical effort and cause large increases in breathing or heart rate’, and the definition of ‘Moderate-intensity activities’ is ‘activities that require moderate physical effort and cause small increases in breathing or heart rate’ [29].

According to the 7th Korea National Health and Nutrition Examination Survey guidelines, walking time was calculated as ‘the number of days walked for at least 10 min at a time during the past week × number of walking hours per day’ [27].

### 2.4. EuroQol-5 Dimension (EQ-5D)

Subjects’ health-related quality of life was measured using the EuroQol-5 Dimension (EQ-5D) developed by the EuroQol Group [31]. EQ-5D was developed to measure overall health as a tool to measure health-related quality of life. In Lee’s study [32], the validity, convergence, and discriminant validity of EQ-5D were confirmed for the general population according to different groups. In addition, it showed an appropriate level of reliability, suggesting that it is useful as an HRQOL tool [32]. In this study, the values calculated by applying the weighting model of the Korea Centers for Disease Control and Prevention in the National Health and Nutrition Examination Survey were used.

It is composed of five domains that ask about current health status: athletic ability, self-care, daily activities, pain/discomfort, and anxiety/depression. Each question was asked to choose the answer that best describes their current health status among three levels: level 1, no problem at all, level 2, moderate problem, and level 3, serious problem. The EQ-5D is a generative variable and it was created by the study of estimating health status and quality of life in the EQ-5D health status.

## 3. Statistical Analysis

In order to present a reliable analysis result representing the population of the Republic of Korea, the final weights suggested by the Korea Centers for Disease Control and Prevention were applied and analyzed. The National Health and Nutrition Examination Survey recommends using a complex sampling design. We used an open-source statistical software, R, version 3.6.1(R Foundation, Vienna, Austria) for all statistical analyses. Descriptive frequencies analysis was conducted according to general characteristics, and BMI × SBR. An independent t-test and analysis of variance (ANOVA) were conducted to compare the analysis of physical activity according to general characteristics, and BMI × SBR. The Scheffe test was conducted for post-hoc analysis. A chi-square test was conducted for the EQ-5D according to physical activity by BMI × SBR. Multiple regression analysis was performed to identify factors affecting health-related quality of life (EQ-5D) according to body type recognition.

## 4. Results

### 4.1. General Characteristics According to Vigorous-Intensity Physical Activity

Vigorous-intensity activity (min) according to the general characteristics was significant according to sex (mean ± SD; male: 10.62 ± 2.56 min and female: 3.31 ± 15.14 min, t = 19.92, *p* < 0.001). According to age groups, it was 9.95 ± 29.97 min for 20–39 year, 7.08 ± 25.50 min for 40–64 year, and 1.41 ± 11.58 min for ≥65 year groups (F = 182.35, *p* < 0.001). Further, according to the post hoc Scheffe test, it was shorter in the 40–64 year age group than the 20–29 year age group and in the ≥65 year age group compared to the 40–64 year age group. Further, according to household income, it was 9.92 ± 29.46 min in the high group, 7.52 ± 26.83 min in the middle-high, 4.45 ± 19.69 min in the middle-low income, and 2.20 ± 15.63 min in the low-income group (F = 98.32, *p* < 0.001). Further, according to the post hoc results, it was shorter in the middle-high income than high, and in the low-income group than middle-low group. According to education levels, it was 9.62 ± 29.10 min for ≥college, 7.71 ± 26.09 min for high school, 3.40 ± 19.33 min for middle school, and 1.04 ± 12.00 min for ≤elementary school (F = 138.96, *p* = 0.001). Further, according to the post hoc results, it was shorter in the high school group than ≥college group, middle school group than the high school group, and ≤elementary school group than the middle school group (Table 1).

### 4.2. General Characteristics According to Moderate-Intensity Physical Activity

Moderate-intensity activity (min) according to general characteristics was significant according to sex (male: 16.11 ± 33.94 min and female: 10.27 ± 25.33 min, t = 13.81, *p* < 0.001). According to age groups, it was 14.62 ± 30.14 min in the 20–39 year, 14.82 ± 31.91 min in the 40–64 year, and 6.98 ± 22.53 min in the ≥65 year group (F = 142.24, *p* < 0.001). Further, post hoc test results showed that it was shorter in the ≥65 year group than 20–29 year and 40–64 year groups. According to household income, it was 17.73 ± 32.56 min, 14.13 ± 30.88 min, 10.33 ± 27.35 min, and 6.64 ± 22.91 min in the high, middle-high, middle-low, and low groups, respectively (F = 138.09, *p* < 0.001). Further, according to the post hoc test, it was shorter in the middle-high group than high group, middle-low group than middle-high group, and low group than middle-low group. According to education levels, it was 17.28 ± 31.62 min, 14.35 ± 31.65 min, 9.9 ± 28.92 min, and 4.67 ± 19.57 min in ≥college, high school, middle school, and ≤elementary school, respectively (F = 195.67, *p* < 0.001). Further, according to the post hoc results, it was shorter in high school than ≥college, middle school than high school, and ≤elementary school than middle school groups (Table 1).

### 4.3. General Characteristics According to Walking

Walking (min) according to general characteristics was significant according to sex (male: 53.92 ± 67.10 min and female: 50.51 ± 62.96 min, t = 3.77, *p* < 0.001). According to age groups, it was 51.30 ± 58.87 min, 53.59 ± 64.05 min, and 49.75 ± 71.39 min for the 20–39 year, 40–64 year, and ≥65 year groups, respectively (F = 6.64, *p* = 0.001). According to the post hoc results, it was shorter in the ≥65 year group than 40–64 year group. According to household income, it was 53.20 ± 59.43 min, 53.06 ± 60.94 min, 52.46 ± 68.19 min, and 47.90 ± 72.19 min for the high, middle-high, middle-low, and low groups, respectively (F = 6.73, *p* < 0.001). According to the post-hoc tests, it was shorter in the low group than high, middle-high, and middle-low groups. According to education levels, it was 52.11 ± 56.34 min, 54.37 ± 63.18 min, 55.00 ± 68.51 min, and 46.83 ± 75.86 min in the ≥college, high school, middle school, and ≤elementary school groups, respectively (F = 14.81, *p* < 0.001). According to the post hoc tests, it was shorter in the elementary school group than ≥college, high school, and middle school groups (Table 1).

### 4.4. Comparison of Vigorous-Intensity Activities Time, Moderate-Intensity Activities Time, and Walking Time According to Difference BMI and Subjective Body Perception

Vigorous-intensity, moderate-intensity, and walking times were compared according to the difference between the BMI and subjective body recognition. In the vigorous-intensity time, BMI and subjective body recognition showed the highest activity time in the same group (6.85 ± 25.20 min). There was a significant difference between vigorous-intensity times between the groups (F = 21.25, *p* = 0.003). Similarly, in the moderate-intensity time, the group with the same BMI and subjective body recognition showed the highest activity time (13.41 ± 30.01 min). There was also a significant difference between moderate-intensity times between the groups (F = 17.24, *p* < 0.001). In the walking time, the group with SBR higher than BMI showed more activity time than the group with BMI higher than SBR (53.04 ± 62.19 min). There was also a significant difference between the groups in walking time (F = 28.32, *p* = 0.034) (Table 2).

### 4.5. Comparison of Exercise Time in Each Body Image Perception and Actual Weight Status Group According to Exercise Intensity

BMI (normal) × SBR (normal) showed the highest vigorous-intensity activity time (7.20 ± 26.05 min), followed by BMI (obesity) × SBR (obesity) (6.93 ± 25.20 min), and BMI (normal) × SBR (underweight) (6.09 ± 24.59 min). In the same exercise intensity, BMI (obesity) × SBR (underweight) showed the lowest score (1.33 ± 7.67 min). As a result of the Scheffe test, a significant difference between the vigorous-intensity activity time for each category was found (F = 37.86, *p* < 0.001) (Table 3).

BMI (normal) × SBR (normal) showed the highest score in moderate-intensity time (13.89 ± 30.18 min), followed by BMI (obesity) × SMB (obesity) (13.37 ±30.22 min). In the same exercise intensity, BMI (obesity) × SBR (underweight) showed the lowest score (4.72 ± 18.43 min). As a result of the Scheffe test, a significant difference between the moderate-intensity activity time for each category was found (F = 43.27, *p* < 0.001). In walking, no significant difference was found for each group (F = 13.77, *p* = 0.197) (Table 3).

### 4.6. Comparison of Health-Related Quality of Life According to Difference BMI and Subjective Body Recognition

For EQ-5D mobility, the normal and abnormal groups had 18,215 (85.5%) and 3111 (14.6%) people, respectively. In the analysis corresponding to the EQ-5D normal, the BMI (normal) × SBR (normal) group had the highest percentage of responses that there was no problem in performing mobility in daily life (n = 6521, 30.6%). The next highest rankings were BMI (obesity) × SBR (obesity) (n = 4924, 23.1%) and BMI (obesity) × SBR (normal) (n = 2874, 13.5%) in order. The groups that corresponded to “abnormal” mobility were BMI (obesity) × SBR (obesity) (n = 1026, 4.8%) and BMI (normal) × SBR (normal) (n = 845, 4.0%) in order. It was confirmed that there was a significant difference for each group regarding mobility (*x*^2^ = 316.38, *p* < 0.001) (Table 4).

For the EQ-5D self-care, there were 20,472 (96.0%) and 854 people (3.9%) in the normal and abnormal groups, respectively. In the analysis corresponding to EQ-5D normal, the BMI (normal) × SBR (normal) group had the highest percentage of responses that there was no problem in performing self-care in daily life (n = 7134, 33.5%). The next highest were BMI (obesity) × SBR (obesity) (n = 5681, 26.6%) and BMI (obesity) × SBR (normal) (n = 3128, 14.7%) in order. The groups that corresponded to “abnormal” were BMI (obesity) × SBR (obesity) (n = 269, 1.3%) and BMI (normal) × SBR (normal) (n = 232, 1.1%) in order. It was confirmed that there was a significant difference for each group regarding self-care (*x*^2^ = 116.36, *p* < 0.001) (Table 4).

For the EQ-5D usual activities, there were 19,536 (91.5%) and 1790 (8.5%) people in the normal and abnormal groups, respectively. In the analysis corresponding to the EQ-5D normal, the BMI (normal) × SBR (normal) group had the highest percentage of responses that there was no problem in performing daily activities (n = 6886, 32.3%). This was followed by BMI (obesity) × SBR (obesity) (n = 5350, 25.1%) and BMI (obesity) × SBR (normal) (n = 3025, 14.2%) in order. The groups that corresponded to “abnormal” of the usual activities were BMI (obesity) × SBR (obesity) (n = 600, 2.8%) and BMI (normal) × SBR (normal) (n = 480, 2.3%) in order. It was confirmed that there was a significant difference for each group regarding user activities (*x*^2^ = 173.75, *p* < 0.001) (Table 4).

For the EQ-5D pain/discomfort, there were 16,258 (76.3%) and 5068 (23.7%) people in the normal and abnormal groups, respectively. As for the analysis corresponding to the EQ-5D normal, the BMI (normal) × SBR (normal) group had the highest percentage of responses that there was no problem with pain-discomfort in daily life (n = 5844, 27.4%). This was followed by BMI (obesity) × SBR (obesity) (n = 4367, 20.5%), BMI (obesity) × SBR (normal) (n = 2427, 11.4%) in order. The groups that corresponded to “abnormal” were BMI (obesity) × SBR (obesity) (n = 1583, 7.4%) and BMI (normal) × SBR (normal) (n = 1522, 7.1%) in order. It was confirmed that there was a significant difference for each group regarding pain/discomfort (*x*^2^ = 80.03, *p* < 0.001) (Table 4).

For the EQ-5D anxiety/depression, there were 19,084 (89.6%) and 2242 (10.4%) people in the normal and abnormal groups, respectively. In the analysis corresponding to the EQ-5D normal, the BMI (normal) × SBR (normal) group had the highest percentage of responses that there was no problem with anxiety/depression in daily life (n = 6726, 31.5%). This was followed by BMI (obesity) × SBR (obesity) (n = 5301, 24.9%) and BMI (obesity) × SBR (normal) (n = 2853, 13.4%) in order. The groups that corresponded “abnormal” were BMI (obesity) × SBR (obesity) (n = 649, 3.0%) and BMI (normal) × SBR (normal) (n = 640, 3.0%) in order. It was confirmed that there was a significant difference for each group regarding anxiety/depression (*x*^2^ = 76.57, *p* < 0.001) (Table 4).

### 4.7. Comparison of EQ-5D Index in Each Body Image Perception and Actual Weight Status Group

The EQ-5D index was analyzed to measure the health-related quality of life of each BMI × SBR group. The BMI < SBR group showed the highest index score (0.95 ± 0.10 min), followed by the BMI = SBR group (0.94 ± 0.12 min). The BMI > SBR group showed the lowest index score (0.92 ± 0.14 min). As a result of the Scheffe test, a significant difference between each BMI × SBR group was found (F = 56.83, *p* < 0.001) (Table 5).

### 4.8. Factors Affecting Health-Related Quality of Life According to BMI and Subjective Body Reception with Multiple Regression Analysis

Factors influencing the health-related quality of life of the group whose ‘BMI = SBR’ were identified through multiple regression analysis. As a result of the regression analysis, the regression model was found to be significant (F = 396.67, *p* < 0.001), and the R^2^ coefficient indicating the explanatory power of the model was 0.165. In this group, the factor that had the greatest influence on health-related quality of life was educational level (β = 0.161), followed by age (β = 0.151) and household income (β = 0.135). As for the effect of PA, it was found that as the vigorous-intensity time, moderate-intensity time, and walking time all increased with a positive sign (+), the health-related quality of life was positively affected (Table 6).

The regression model of the ‘BMI < SBR’ group was found to be significant (F = 73.01, *p* < 0.001), and the R^2^ coefficient of the model was 0.132. The major factors affecting the health-related quality of life in this group were the education level (β = 0.178) and age (β = −0169) in order. As an influencing factor according to PA, only walking time was found to positively affect the health-related quality of life as the positive sign (+) increased (Table 6).

The regression model of the ‘BMI > SBR’ group was found to be significant (F = 135.12, *p* < 0.001), and the R^2^ coefficient of the model was 0.192. The major factors affecting the health-related quality of life in this group were age (β = −0.186), household income (β = 0.144), and walking time (β = 0.126), in order (Table 6).

## 5. Discussion

This study analyzed the physical activity and quality of life according to the match between the actual degree of obesity (BMI) and an individual’s subjective body perception for adults over 20 years.

In this study, the 20–39 year age group showed the highest amount of vigorous-intensity physical activity. The highest amount of physical activity in the group ‘vigorous’ was also confirmed in the study by Al-Hazzaa et al. [33]. Further, the rate of vigorous-intensity physical activity was the highest among 15–44 year. In contrast, regarding physical activity according to gender, it was confirmed that women performed a higher amount of activity than men, which indicated a difference from our study.

In the difference in the amount of activity according to academic background, university or higher graduates showed the highest amount of vigorous and moderate intensity physical activity than other groups. It was found that the higher the educational level, the higher the activity of vigorous-intensity and moderate-intensity physical activity (*p* < 0.001). In the study by Macel et al. [34], the education level was divided into lower (primary or vocational) and upper levels (secondary or higher). Similar to our study, at a moderate level of physical activity, it was found that as the educational level increased, the amount of activity increased. It was found that activity increased with age (OR: 1.25, 95% CI: 1.06–1.48). In contrast, the opposite result was found at a vigorous level of physical activity (OR: 0.84 95% CI: 0.72–0.99).

Examining the physical activity according to the difference between the actual degree of obesity and body-type perception, the group with the matched actual degree of obesity and subjectively felt body perception had the highest amount of vigorous- and moderate-intensity physical activity (vigorous-intensity: *p* = 0.003 and moderate-intensity: *p* < 0.001). It was confirmed that the BMI = normal × SBR = normal group had the most physical activity among the groups in which the BMI and SBR matched (vigorous-intensity: 7.20 ± 26.05 min and moderate intensity: 13.89 ± 30.18 min).

In the group with high SBR, psychological distress occurred as social rejection and internalization of weight stigma were formed. Negative emotion and psychological distress lead to a common coping response, such as comfort eating. In this emotional state, it is difficult to achieve long-term weight loss. Even weight loss requires psychological resources for healthier eating and physical activity. However, these are damaged by psychological distress and interfere with weight loss. In this study, the result of lower physical activity in the BMI < SBR group than in the BMI = SBR group was also considered to be caused by psychological distress [35].

In a study on the Canadian adult population [36], according to the actual BMI and body type perceived subjectively, the subjectively perceived health status was compared. In this study, as the distorted perception of weight increased, the subjectively perceived health of “fair” was 1.16 times higher than that of the matching group (healthy weight-about right) [36]. Furthermore, regarding the subjective quality of life, the distorted perception of weight gets bigger the higher the rate of response of unsatisfied life satisfaction (1.22 times; 95% CI: 1.10–1.35). Although both perceived weight status and actual weight status affected self-assessed health and life satisfaction, it was suggested that perceived weight status had a higher influence on an individual’s subjective health and quality of life. Since there is a high correlation between weight loss attempts and health promotion behaviors according to body-type recognition, it is important to correctly recognize one’s actual body type. According to the 8th National Health and Nutrition Examination Survey of 2019, the prevalence of obesity in the entire adult population in Korea increased from 26% in 1998 to 30.7% in 2008 and 33.8% in 2019, which showed an increase of 7.8% over the past 20 years [37]. As the prevalence of chronic diseases is also increasing due to an increase in the prevalence of obesity [37], health promotions through maintaining a normal weight are emerging as a social concern. Hence, related policy suggestions and establishments are required [38].

However, often one does not know the exact degree of one’s actual obesity, including weight. In fact, according to a telephone survey of adults aged 19 years or older enrolled in health insurance and over normal weight (18 ≤ BMI), 79.5% perceived obesity as a serious problem, and 60.5% perceived their body shape as “very overweight or overweight.” However, in the normal and overweight (18 ≤ BMI < 25) group, 33.4% perceived themselves as lean or fat rather than a normal body type, and approximately 18% in the obese and highly obese (25 ≤ BMI) group regarded themselves as average. [38].

The National Health Plan 2030 suggests mid- to long-term policy directions for disease prevention and health promotion and efficient operation and goal achievements of the National Health Promotion Comprehensive Plan through performance index monitoring and evaluation [39]. It has secured health equity among genders, classes, and regions with the vision of “a society where everyone can enjoy lifelong health” and expanded the scope of application to all people. In addition, for a society that enjoys lifelong health, it covers everyone, including the government, guaranteeing the right to health throughout one’s lifespan. Prevention of non-infectious diseases, classified as a priority, includes cancer, cardiovascular disease, injury, and obesity.

As a task for obesity, the establishment of an integrated governance and environment for obesity prevention was selected. For this, obesity prevention, provision of management services, establishment of a foundation for managing severe obesity, and improvement of obesity-inducing environment and lifestyles were included as detailed tasks. As the first step, it is important to accurately understand one’s BMI, an objective indicator other than one’s SBR. BMI is an indicator that can easily measure anyone. Further, it is the correct recognition of one’s body type, which creates an appropriate psychological emotion, thereby creating a virtuous cycle of weight control. The importance of maintaining a normal weight and nutrition education on desirable weight control methods should be followed to lay the foundation for implementing a health plan that “everyone enjoys lifelong health.”

This study has some limitations. First, it was a cross-sectional study that used data from the National Health and Nutrition Examination Survey. Hence, it was not possible to confirm the causal relationship between the actual degree of obesity and the inconsistency in body shape, and the resulting physical activity and quality of life. Second, there was a limit to the measurement since the physical activity, a health promotion activity, was identified in the form of a self-reported questionnaire using the recall method for the past week. It was difficult to properly reflect the qualitative aspect of the health promotion activity. For these points, further investigation on the relationship between health promotion behaviors through supplementation of related research results and the implementation of health promotion programs is required.

The importance of one’s health awareness and health information comprehension is emerging for the improvement of universal health levels and health equity. Hence, to prevent excessive weight control behavior due to incorrect body-type recognition, nutrition education should be the basis for correct body-type recognition. In addition, this should be accompanied by the importance of maintaining a normal weight, proper weight control, and health promotion programs for correct body-type recognition.

## 6. Conclusions

This study confirmed the difference in physical activity time and health-related quality of life according to the distorted perception of weight for the general population of the National Health and Nutrition Examination Survey. Vigorous-intensity and moderate-intensity activity times were found to be greater in the group with no distorted perception of weight. It was found that the group with normal BMI and subjective body recognition showed the most active time. This group had the highest percentage of responding as normal in the five items of the EQ-5D, which indicated the health-related quality of life. Hence, it was confirmed that various health promotion programs are required to narrow the gap between subjective body perception as well as actual obesity management. Therefore, the establishment of multi-sectoral policies is necessary.

## Figures and Tables

**Table 1:** General characteristics according to vigorous-intensity, moderate-intensity, and walking time.

		Vigorous-Intensity		Moderate-Intensity		Walking	
Variables	n (%)	Mean ± SD (min)	t or F(*p*) Scheffe	Mean ± SD (min)	t or F(*p*) Scheffe	Mean ± SD (min)	t or F(*p*) Scheffe
Sex							
Male	9153 (42.9)	10.62 ± 2.56	19.921 (<0.001)	16.11 ± 33.94	13.815 (<0.001)	53.92 ± 67.10	3.775 (<0.001)
Female	12,173 (57.1)	3.31 ± 15.14		10.27 ± 25.33		50.51 ± 62.69	
Age							
20–39 ^a^	5986 (28.1)	9.95 ± 29.97	182.351 (<0.001) c < b < a	14.62 ± 30.14	142.245 (<0.001) c < a,b	51.30 ± 58.87	6.643 (0.001) c < b
40–64 ^b^	9927 (46.5)	7.08 ± 25.50		14.82 ± 31.91		53.59 ± 64.05	
≥65 ^c^	5413 (25.4)	1.41 ± 11.58		6.98 ± 22.53		49.75 ± 71.39	
Household income							
High ^a^	6114 (28.7)	9.92 ± 29.46	98.323 (<0.001) d < c < b < a	17.73 ± 32.56	138.096 (<0.001) d < c < b < a	53.20 ± 59.43	6.731 (<0.001) d < a,b,c
Middle-high ^b^	5939 (27.8)	7.52 ± 26.83		14.13 ± 30.88		53.06 ± 60.94	
Middle-low ^c^	5233 (24.5)	4.45 ± 19.69		10.33 ± 27.35		52.46 ± 68.19	
Low ^d^	4040 (18.9)	2.20 ± 15.63		6.46 ± 22.91		47.90 ± 72.19	
Education level							
≥College ^a^	7460 (35.0)	9.62 ± 29.10	138.969 (<0.001) d < c < b < a	17.28 ± 31.62	195.670 (<0.001) d < c < b < a	52.11 ± 56.34	14.815 (<0.001) d < a,b,c
High school ^b^	6896 (32.3)	7.71 ± 26.09		14.35 ± 31.65		54.37 ± 63.18	
Middle school ^c^	2245 (10.5)	3.40 ± 19.33		9.99 ± 28.92		55.00 ± 68.51	
≤Elementary school ^d^	4725 (22.2)	1.04 ± 12.00		4.67 ± 19.57		46.83 ± 75.86	
Total	21,326(100.0)						

Note. The definition of ‘vigorous-intensity’ is ‘activities that require hard physical effort and cause large increases in breathing or heart rate’ and ‘moderate-intensity’ is activities that require moderated physical effort and cause small increases in breathing or heart rate. BMI = body mass index, SBR = subjective body recognition, SD = standard deviation, Min = minutes.

**Table 2:** Comparison of vigorous-intensity activities time, moderate-intensity activities time, and walking time according to difference between BMI and subjective body perception.

Groups	n (%)	Vigorous-Intensity		Moderate-Intensity		Walking	
		Mean ± SD (min)	F(*p*) *p*-Value Scheffe	Mean ± SD (min)	F(*p*) *p*-Value Scheffe	Mean ± SD (min)	F(*p*) *p*-Value Scheffe
BMI = SBR ^a^	14,060 (65.9)	6.85 ± 25.20	21.25 (0.003) b < a	13.41 ± 30.01	17.24 (0.001) c < a,b	52.38 ± 63.83	28.32 (0.034) C < b
BMI < SBR ^b^	3319 (15.6)	5.48 ± 21.73		12.78 ± 28.57		53.04 ± 62.19	
BMI > SBR ^c^	3947 (18.5)	5.81 ± 23.99		10.49 ± 28.19		49.60 ± 69.33	
Total	21,326 (100.0)	6.44 ± 24.47		12.77 ± 29.48		51.97 ± 64.64	

Note. The definition of ‘vigorous-intensity’ is ‘activities that require hard physical effort and cause large increases in breathing or heart rate’ and ‘moderate-intensity’ is ‘activities that require moderated physical effort and cause small increases in breathing or heart rate’. BMI = body mass index, SBR = subjective body recognition, SD = standard deviation, min = minutes.

**Table 3:** Comparison of exercise time in each body image perception and actual weight status group according to exercise intensity.

			Vigorous-Intensity		Moderate-Intensity		Walking	
Groups	Sub-Groups	n (%)	Mean ± SD (min)	F(*p*) *p*-Value Duncan	Mean ± SD (min)	F(*p*) *p*-Value Duncan	Mean ± SD (min)	F(*p*) *p*-Value Duncan
BMI = SBR	BMI (normal) × SBR (normal) ^a^	7366 (34.5)	7.20 ± 26.05	37.86 (<0.001)	13.89 ± 30.18	43.27 (<0.001) (f,h < a,b)	52.70 ± 63.33	13.77 (0.197)
	BMI (obesity) × SBR (obesity) ^b^	5950 (27.9)	6.93 ± 25.20		13.37 ± 30.22		52.33 ± 63.48	
	BMI (underweight) × SBR (underweight) ^c^	744 (3.5)	2.78 ± 13.58		9.01 ± 26.01		49.74 ± 71.15	
BMI < SBR	BMI (normal) × SBR (obesity) ^d^	3199 (15.0)	5.50 ± 21.83		13.01 ± 28.85		53.16 ± 62.56	
	BMI (underweight) × SBR (obesity) ^e^	5 (0.0)	0.00 ± 0.00		0.00 ± 0.00		92.00 ± 118.20	
	BMI (underweight) × SBR (normal) ^f^	115 (0.5)	5.30 ± 19.30		7.17 ± 19.47		48.04 ± 47.02	
BMI > SBR	BMI (normal) × SBR (underweight) ^g^	2660 (12.5)	6.09 ± 24.59		11.24 ± 29.19		49.23 ± 69.66	
	BMI (obesity) × SBR (normal) ^h^	1197 (5.6)	5.54 ± 23.42		9.25 ± 26.39		50.53 ± 67.83	
	BMI (obesity) × SBR (underweight) ^i^	90 (0.4)	1.33 ± 7.67		4.72 ± 18.43		48.39 ± 79.42	
Total	Total	21,326 (100.0)	6.45 ± 24.47		12.77 ± 29.48		51.97 ± 64.64	

Note. The definition of ‘vigorous-intensity’ is ‘activities that require hard physical effort and cause large increases in breathing or heart rate’ and ‘moderate-intensity’ is ‘activities that require moderated physical effort and cause small increases in breathing or heart rate’. BMI = body mass index, SBR = subjective body recognition.

**Table 4:** Comparison of health-related quality of life according to difference between BMI and subjective body recognition.

Variables	Groups	Groups	Normal		Abnormal		*x* ^2^	*p*-Value
			Frequency	%	Frequency	%		
Mobility	BMI = SBR	BMI (normal) × SBR (normal) ^e^	6521	30.6%	845	4.0%	316.38	<0.001
		BMI (obesity) × SBR (obesity) ^g^	4924	23.1%	1026	4.8%		
		BMI (underweight) × SBR (underweight) ^c^	639	3.0%	105	0.5%		
	BMI < SBR	BMI (normal) × SBR (obesity) ^d^	899	4.2%	298	1.4%		
		BMI (underweight) × SBR (obesity) ^a^	54	0.3%	36	0.2%		
		BMI (underweight) × SBR (normal) ^b^	2192	10.3%	468	2.2%		
	BMI > SBR	BMI (normal) × SBR (underweight)f	108	0.5%	7	0.0%		
		BMI (obesity) × SBR (normal) ^h^	2874	13.5%	325	1.5%		
		BMI (obesity) × SBR (underweight) ^i^	4	0.0%	1	0.0%		
Total			18,215	85.5%	3111	14.6%		
Self-care	BMI = SBR	BMI (normal) × SBR (normal) ^e^	7134	33.5%	232	1.1%	116.36	<0.001
		BMI (obesity) × SBR (obesity) ^g^	5681	26.6%	269	1.3%		
		BMI (underweight) × SBR (underweight) ^c^	701	3.3%	43	0.2%		
	BMI < SBR	BMI (normal) × SBR (obesity) ^d^	1103	5.2%	94	0.4%		
		BMI (underweight) × SBR (obesity) ^a^	81	0.4%	9	0.0%		
		BMI (underweight) × SBR (normal) ^b^	2525	11.8%	135	0.6%		
	BMI > SBR	BMI (normal) × SBR (underweight) ^f^	114	0.5%	1	0.0%		
		BMI (obesity) × SBR (normal) ^h^	3128	14.7%	71	0.3%		
		BMI (obesity) × SBR (underweight) ^i^	5	0.0%	0	0.0%		
Total			20,472	96.0%	854	3.9%		
Usual activities	BMI = SBR	BMI (normal) × SBR (normal) ^e^	6886	32.3%	480	2.3%	173.75	<0.001
		BMI (obesity) × SBR (obesity) ^g^	5350	25.1%	600	2.8%		
		BMI (underweight) × SBR (underweight) ^c^	664	3.1%	80	0.4%		
	BMI < SBR	BMI (normal) × SBR (obesity) ^d^	1033	4.8%	164	0.8%		
		BMI (underweight) × SBR (obesity) ^a^	72	0.3%	18	0.1%		
		BMI (underweight) × SBR (normal) ^b^	2390	11.2%	270	1.3%		
	BMI > SBR	BMI (normal) × SBR (underweight) ^f^	112	0.5%	3	0.0%		
		BMI (obesity) × SBR (normal) ^h^	3025	14.2%	174	0.8%		
		BMI (obesity) × SBR (underweight) ^i^	4	0.0%	1	0.0%		
Total			19,536	91.5%	1790	8.5%		
Pain/ Discomfort	BMI = SBR	BMI (normal) × SBR (normal) ^e^	5844	27.4%	1522	7.1%	80.03	<0.001
		BMI (obesity) × SBR (obesity) ^g^	4367	20.5%	1583	7.4%		
		BMI (underweight) × SBR (underweight) ^c^	559	2.6%	185	0.9%		
	BMI < SBR	BMI (normal) × SBR (obesity) ^d^	885	4.1%	312	1.5%		
		BMI (underweight) × SBR (obesity) ^a^	59	0.3%	31	0.1%		
		BMI (underweight) × SBR (normal) ^b^	2016	9.5%	644	3.0%		
	BMI > SBR	BMI (normal) × SBR (underweight) ^f^	97	0.5%	18	0.1%		
		BMI (obesity) × SBR (normal) ^h^	2427	11.4%	772	3.6%		
		BMI (obesity) × SBR (underweight) ^i^	4	0.0%	1	0.0%		
Total			16,258	76.3%	5068	23.7%		
Anxiety/Depression	BMI = SBR	BMI (normal) × SBR (normal) ^e^	6726	31.5%	640	3.0%	76.57	<0.001
		BMI (obesity) × SBR (obesity) ^g^	5301	24.9%	649	3.0%		
		BMI (underweight) × SBR (underweight) ^c^	624	2.9%	120	0.6%		
	BMI < SBR	BMI (normal) × SBR (obesity) ^d^	1080	5.1%	117	0.5%		
		BMI (underweight) × SBR (obesity) ^a^	77	0.4%	13	0.1%		
		BMI (underweight) × SBR (normal) ^b^	2315	10.9%	345	1.6%		
	BMI > SBR	BMI (normal) × SBR (underweight) ^f^	105	0.5%	10	0.0%		
		BMI (obesity) × SBR (normal) ^h^	2853	13.4%	346	1.6%		
		BMI (obesity) × SBR (underweight) ^i^	3	0.0%	2	0.0%		
Total			19,084	89.6%	2242	10.4%		

Note. BMI = body mass index, SBR = subjective body recognition.

**Table 5:** Comparison of EQ-5D index in each body image perception and actual weight status group.

Groups	n (%)	EQ-5D Index		
		Mean ± SD	F	*p*-Value Scheffe
BMI = SBR ^a^	14,060 (65.9)	0.94 ± 0.12	56.83	<0.001
BMI < SBR ^b^	3319 (15.6)	0.95 ± 0.10		c < a < b
BMI > SBR ^c^	3947 (18.5)	0.92 ± 0.14		

Note. EQ-5D = Euro-Quality of Life 5 Dimension, BMI = body mass index, SBR = subjective body recognition, SD = standard deviation.

**Table 6:** Factors affecting health-related quality of life according to BMI and subjective body reception with multiple regression analysis.

Variables	BMI = SBR				BMI < SBR				BMI > SBR			
	B	S.E.	β	t (*p*)	B	S.E.	β	t (*p*)	B	S.E.	β	t (*p*)
(Constants)	4.155	0.068		60.77 ***	4.359	0.139		31.41 ***	4.422	0.141		3.28 ***
Sex: Female (Ref: Male) ^☨^	−0.191	0.018	−0.085	−10.75 ***	−0.176	0.039	−0.075	−4.55 ***	−0.233	0.039	−0.091	−6.00 ***
Age	−0.010	0.001	−0.151	−15.60 ***	−0.011	0.001	−0.169	−8.65 ***	−0.014	0.001	−0.186	−9.80 ***
Household income	0.139	0.009	0.135	15.43 ***	0.093	0.017	0.098	5.49 ***	0.163	0.019	0.144	8.38 ***
Education level	0.158	0.010	0.161	15.46 ***	0.174	0.020	0.178	8.70 ***	0.109	0.021	0.104	5.10 ***
PA: Vigorous- intensity	0.003	0.001	0.042	5.01 ***	0.001	0.001	0.021	1.17	0.002	0.001	0.025	1.64
PA: Moderate-intensity	0.003	0.000	0.053	6.14 ***	0.001	0.001	0.026	1.43	0.004	0.001	0.075	4.82 ***
PA: Walking	0.003	0.000	0101	11.22 ***	0.002	0.000	0.064	3.33 **	0.004	0.000	0.126	8.08 ***
F(*p*)	396.57 (<0.001)				73.01 (<0.001)				135.12 (<0.001)			
R^2^	0.165				0.134				0.194			
Adj. R^2^	0.165				0.132				0.192			

Note. Except for ‘Sex’, age, household income, education level, PA (vigorous-intensity, moderated-intensity, and walk group) variables are analyzed as continuous variables. ^☨^ dummy coded, BMI = body mass index, SBR = subjective body recognition, S.E. = standard error, PA = physical activity. ** *p* < 0.01, *** *p* < 0.001.

## Data Availability

The datasets that were generated and analyzed during the current study are not publicly available but are available from the corresponding author on reasonable request.
